# Characterization of gene regulatory networks underlying key properties in human hematopoietic stem cell ontogeny

**DOI:** 10.1186/s13619-024-00192-z

**Published:** 2024-04-17

**Authors:** Fei Li, Yanling Zhu, Tianyu Wang, Jun Tang, Yuhua Huang, Jiaming Gu, Yuchan Mai, Mingquan Wang, Zhishuai Zhang, Jiaying Ning, Baoqiang Kang, Junwei Wang, Tiancheng Zhou, Yazhou Cui, Guangjin Pan

**Affiliations:** 1 Key Laboratory of Immune Response and Immunotherapy, Joint School of Life Sciences, Guangzhou Institutes of Biomedicine and Health, Chinese Academy of Sciences; Guangzhou Medical University, Guangzhou, 510530 China; 2 University of Chinese Academy of Sciences, Beijing, 100049 China; 3 Centre for Regenerative Medicine and Health, Hong Kong Institute of Science and Innovation, Chinese Academy of Sciences, Hong Kong, China; 4 Guangdong Provincial Key Laboratory of Stem Cell and Regenerative Medicine, Guangdong-Hong Kong Joint Laboratory for Stem Cell and Regenerative Medicine, Center for Cell Lineage and Cell Therapy, Guangzhou Institutes of Biomedicine and Health, Chinese Academy of Sciences, Guangzhou, 510530 China; 5 GIBH-HKU Guangdong-Hong Kong Stem Cell and Regenerative Medicine Research Centre, GIBH-CUHK Joint Research Laboratory On Stem Cell and Regenerative Medicine, Guangzhou Institutes of Biomedicine and Health, Chinese Academy of Sciences, Guangzhou, 510530 China; 6 South China Institute for Stem Cell Biology and Regenerative Medicine, Guangzhou Institutes of Biomedicine and Health, Chinese Academy of Sciences, Guangzhou, 510530 China; 7 Key Lab for Rare & Uncommon Diseases of Shandong Province, Biomedical Sciences College & Shandong Medicinal Biotechnology Centre, Shandong First Medical University & Shandong Academy of Medical Sciences, Ji’nan, 250117 Shandong China

**Keywords:** Hematopoietic stem cell, Transcription factors, Lineage potential, Gene regulatory networks, Human induced pluripotent stem cells, Hematopoietic differentiation

## Abstract

Human hematopoiesis starts at early yolk sac and undergoes site- and stage-specific changes over development. The intrinsic mechanism underlying property changes in hematopoiesis ontogeny remains poorly understood. Here, we analyzed single-cell transcriptome of human primary hematopoietic stem/progenitor cells (HSPCs) at different developmental stages, including yolk-sac (YS), AGM, fetal liver (FL), umbilical cord blood (UCB) and adult peripheral blood (PB) mobilized HSPCs. These stage-specific HSPCs display differential intrinsic properties, such as metabolism, self-renewal, differentiating potentialities etc. We then generated highly co-related gene regulatory network (GRNs) modules underlying the differential HSC key properties. Particularly, we identified GRNs and key regulators controlling lymphoid potentiality, self-renewal as well as aerobic respiration in human HSCs. Introducing selected regulators promotes key HSC functions in HSPCs derived from human pluripotent stem cells. Therefore, GRNs underlying key intrinsic properties of human HSCs provide a valuable guide to generate fully functional HSCs in vitro.

**Supplementary Information:**

The online version contains supplementary material available at 10.1186/s13619-024-00192-z.

## Background

The ontogeny of hematopoiesis is a developmentally dynamic process consisting multiple waves and each wave generates hematopoietic compartment containing specific lineage subsets (Laurenti and Göttgens 2018; Orkin and Zon 2008; Dzierzak and Bigas 2018; Haas et al. 2018; Ivanovs et al. 2017; Copley and Eaves 2013). The first wave of human hematopoiesis occurs in yolk sac (YS) and mainly generates primitive macrophages, erythroid as well as erythromyeloid progenitors (EMPs) (Orkin and Zon 2008; Dzierzak and Bigas 2018; Silver and Palis 1997; McGrath et al. 2015). Lymphoid cells such as NK cells are also reported to be generated in YS hematopoiesis (McGrath et al. 2015; Böiers et al. 2013; Huang et al. 1994; Dege et al. 2020). YS hematopoiesis is considered to be HSC independent as definitive HSCs emerge in the aorta-gonad-mesonephros (AGM) region at later developmental stage (Dzierzak and Bigas 2018; Medvinsky and Dzierzak 1996; Tavian et al. 2001; Oberlin et al. 2002). AGM-HSCs further get functional maturation in fetal liver, the important niche for definitive hematopoiesis during the whole embryonic development (Orkin and Zon 2008; Dzierzak and Bigas 2018; Popescu et al. 2019; Calvanese et al. 2022). In recent years, single cell transcriptome analysis has been applied to map human hematopoiesis at various developmental stages and sites (Popescu et al. 2019; Calvanese et al. 2022; Bian et al. 2020; Ranzoni et al. 2021; Zeng et al. 2019; Liggett and Sankaran 2020). These single cell transcriptome data confirm that the blood/immune lineage composition varies in site- and stage- specific hematopoietic compartment in human ontogeny. In general, early stage hematopoiesis displays erythroid/megakaryocyte (Er/Mk) lineage bias while lymphoid (Ly) and myeloid (My) lineages are more evident at later developmental stages (Copley and Eaves 2013; Popescu et al. 2019; Kashem et al. 2017; Roy et al. 2021), indicating a progressive maturation towards fully functional hematopoiesis over development.

Residing on the top in hematopoiesis hierarchy, human HSCs also show substantial changes in molecular signatures over development. AGM-HSCs exhibit nascent HSC gene signatures and these nascent signatures are suppressed while maturation signatures get acquired in fetal liver (FL) HSCs (Copley and Eaves 2013; Popescu et al. 2019; Calvanese et al. 2022). In addition, low-lineage primed or naïve HSPCs at different developmental stages or sites exhibit differential intrinsic properties, such as self-renewal, differentiating potentialities etc. (Ivanovs et al. 2017; Copley and Eaves 2013; Roy et al. 2021; Mack et al. 2021). For instance, low-primed multipotential progenitors in human YS (YS-MPs) mainly give rise to Er/Mk/My linages and are not known to have long-term repopulating potential (Copley and Eaves 2013; Popescu et al. 2019; Soares-da-Silva et al. 2021; Palis 2016). In contrast, human AGM-HSCs have robust repopulating potency based on immunodeficient mouse model and are much more self-renewal than the umbilical cord-blood (UCB) HSCs (Ivanovs et al. 2011; Notta et al. 2011; Catlin et al. 2011). Human FL-HSCs also show more potency than HSCs from the adult bone marrow (Bowie et al. 2007; Rebel et al. 1996; Holyoake et al. 1999). In mouse studies, the differential properties in fetal and adult HSCs are controlled by differential transcriptional programs. For example, Sox17, Ezh2 are required to maintain fetal and neonatal HSCs, but not adult HSCs (Kim et al. 2007; Mochizuki-Kashio et al. 2011). In contrast, Bmi1, Gfi1, Etv6 and C/EBPa promote the self-renewal of adult HSCs while are not functional in fetal HSCs (Park et al. 2003; Hock et al. 2004a; Hock et al. 2004b; Ye et al. 2013). At the mean time, the gene regulatory networks (GRNs) and the key regulators that control the intrinsic property changes in human HSCs over development remain unclear. To address this issue is critical as re-establishing GRNs controlling key HSC properties would promote the generation of fully functional HSPCs from human pluripotent stem cells (hPSCs).

Here in this study, we analyzed single-cell transcriptome of low-lineage primed or primary HSPCs at different developmental stages and revealed key property changes in human HSC ontogeny. We identified GRNs and their key regulators controlling differential key HSC functions such as self-renewal, lymphoid potency and metabolism etc.. Introducing the selected regulators promotes key functions in HSPCs derived from human induced pluripotent stem cells (hiPSCs).

## Results

### Distinct properties in human less lineage primed HSPCs over development

**Fig. 1:**
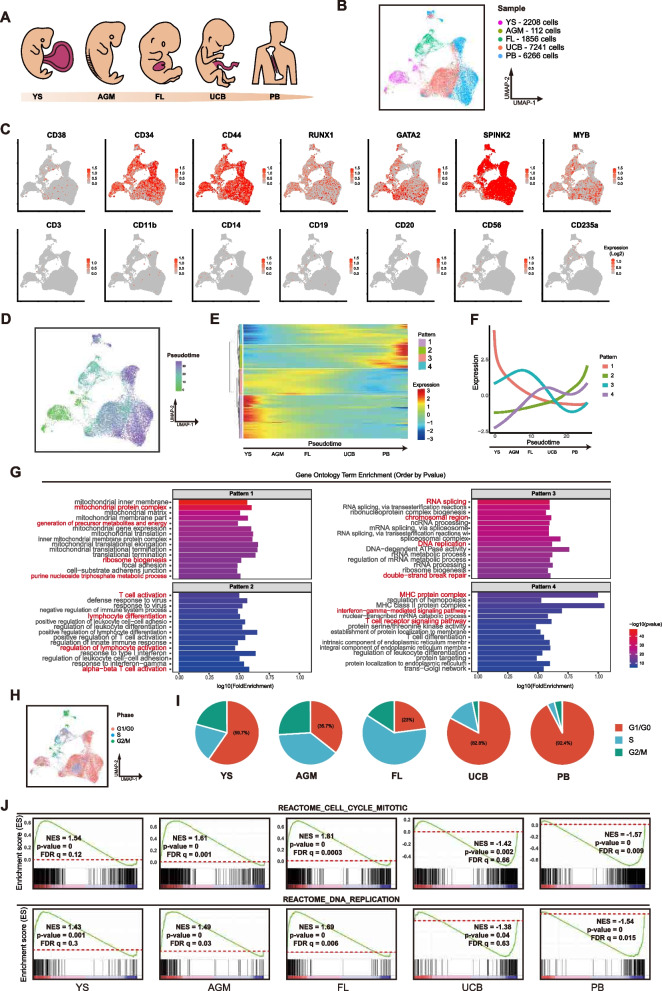
Distinct properties in human HSPCs at different developmental stages.** A** Scheme of analyzed HSPC samples at various stages of human development. The analyzed single cell transcriptome data of CD34 + CD38-Lin- HSPCs are from yolk sac (YS, CS10/CS11)、AGM (CS13), fetal liver (FL, week7-week17), umbilical cord blood (UCB) and mobilized in peripheral blood (PB). **B** Uniform manifold and projection (UMAP) plot of single cell transcriptomes of HSPCs from various stages indicated by different colors.** C** Plots of blood lineage gene expressions in HSPCs at different stages. Color indicate expression level (TPM, Log-scaled). **D** Pseudotime developmental path analysis on HSPCs at different stages. UMAP plot is ordered by pseudotime score as colored.** E** Heatmap for gene clusters with expression changes along the pseudotime progression. Four clusters based on changed pattern are indicated by different colors. The scaled expression level (TPM, z-normalized) of each cluster are shown. **F** Schematic plot of dynamic changes of each cluster genes across pseudotime. Lines indicate mean values of scaled expression levels (TPM, z-normalized). **G** Top Gene Ontology (GO) terms enriched in different gene clusters. Terms of critical HSC functions are highlighted.** H** UMAP plot of predicated cell cycle phases indicted by different colors.** I** Cell cycle phases of HSPCs from different stages.** J** GSEA score plots of the gene sets related to cell cycle and DNA replication in HSPCs at different stages

We sought to analyze and compare the properties of human naïve HSPCs from different developmental stages at single cell level. We downloaded the single-cell transcriptome data of human YS-MP (Bian et al. 2020), AGM-HSCs (Zeng et al. 2019), FL-HSCs (Popescu et al. 2019) and also generated single-cell transcriptome data for the sorted less-lineage primed (LP) HSPCs (CD34^+^/CD38^−^/lineage^−^) either from the umbilical cord-blood (UCB) or mobilized healthy adult peripheral blood (PB). All the single cell data were re-normalized to remove the batch to batch variations (Fig. 1A-B). Generally, the single cell transcriptome data of these LP-HSPCs formed discrete clusters highly related to their developmental stages (Fig. 1B). However, the well-known human HSC markers such as CD34, CD44, RUNX1, GATA2 etc. were uniformly expressed in all LP-HSPCs, including YS-MPs that are usually not known as definitive HSCs (Fig. 1C). As expected, lineage markers were not expressed in all LP-HSPCs (Fig. 1C). We then identified gene clusters that were progressively up- or down-regulated in LP-HSPCs across development by pseudotime analysis (Fig. 1D-F). Genes related to mitochondrial functions, metabolism etc. were highly active in YS-MPs, but rapidly declined in LP-HSPCs at later developmental stages (Fig. 1E-G, pattern1). Genes related to DNA replication, RNA biosynthesis etc. were mostly active in FL-HSCs, but immediately suppressed in UCB- and PB-HSPCs (Fig. 1E-G, pattern 3). Lineage potentiaties, particularly myeloid, lymphoid potency were progressively acquired in HSCs over development and were mostly active in PB-HSPCs (Fig. 1E-G, pattern 2). Major histocompatibility complex (MHC) started to express at FL-HSPCs (Fig. 1E-G, pattern 4). Human LP-HSPCs showed distinct cell cycle states over development (Fig. 1H-I). Generally, the pre-natal HSPCs showed active self-renewal while the postnatal HSCs were quiescent (Fig. 1H-I), consistenting to many other reports (Copley and Eaves 2013; Popescu et al. 2019; Calvanese et al. 2022; Roy et al. 2021). Gene Set Enrichment Analysis (GSEA) further revealed that the mitotic, DNA replication and proliferation were substantially active in prenatal, but inactive in postnatal HSPCs (Fig. 1J). Together, single-cell transcriptome data show that human low lineage primed HSPCs undergo intrinsic changes in key properties over development.

### Metabolism features in human less lineage primed HSPCs over development

**Fig. 2:**
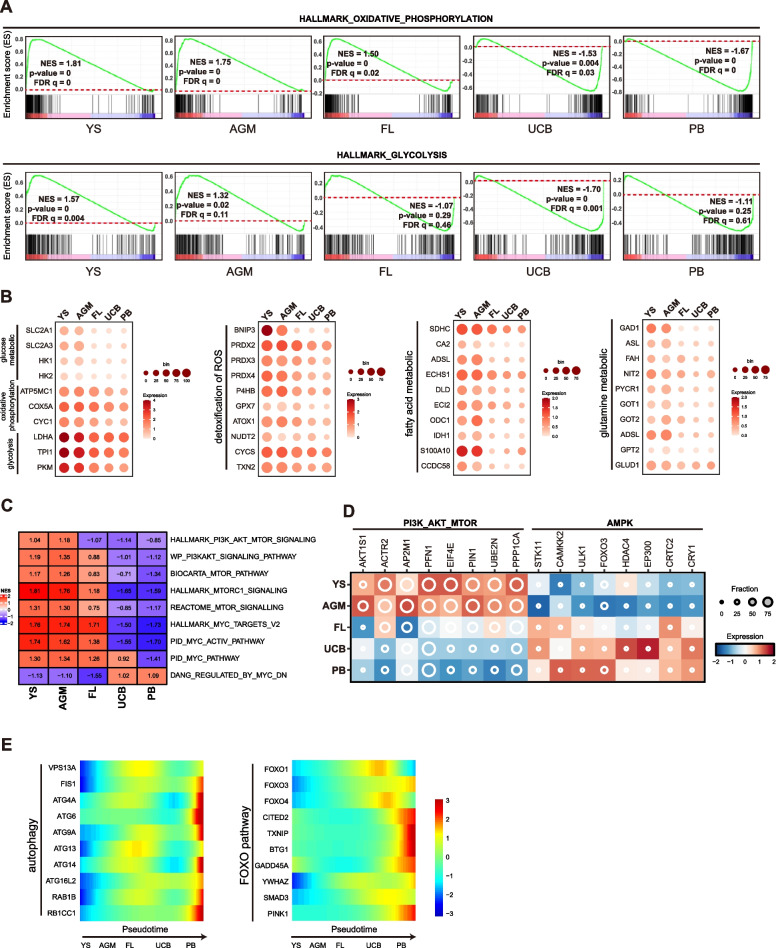
Metabolism features in human HSPCs at different developmental stages. **A** GSEA score plots of the gene sets with oxidative phosphorylation or glycolysis in HSPCs at different stages.** B** Dot plot of the expression level of selected metabolism genes in different HSPCs. Color indicates the average of standardized gene expression (TPM, Log-scaled) and size of the dot indicates the percentage of cells expressing this gene in each group.** C** Heatmap of GSEA score in different HSPCs for the indicated gene sets. GSEA scores indicated by NES number are shown and differentially colored. **D** Dot plot displaying the expression level of selected genes involved in PI3K-AKT-MTOR and AMPK pathway in different HSPCs. Color indicates the scaled mean expression level (TPM, Log-scaled) and the size of the dot indicates the percentage of cells expressing the given gene.** E** Heatmap for expressions of gene clusters related to autophagy and FOXO pathway along the pseudotime progression. Color displays the scaled expression level (TPM, z-normalized)

Metabolism plays important roles to maintain normal functions of HSPCs. Human LP-HSPCs from different developmental stages displayed distinct metabolism features (Fig. 2A-B). In general, the pre-natal HSPCs showed much higher metabolic activities than that of post-natal HSCs, such as glucose, fatty acid glutamine metabolic etc. (Fig. 2A-B). In particular, YS-MPs, AGM-HSCs displayed higher level of glucose metabolic activities in both oxidative phosphorylation (OXPHOS) and glycolysis (Fig. 2A). The UCB- and PB-HSPCs showed much reduced OXPHOS and mainly rely on glycolysis for glucose metabolism (Fig. 2A-B). Reactive oxygen species (ROS) metabolic was active in all stage HSPCs in development, albeit is relatively higher in pre-natal HSPCs (Fig. 2B). The HSPCs at early developmental stages also showed higher fatty acid and glutamine metabolic than UCB- and PB-HSPCs (Fig. 2B). PI3K-AKT-mTOR and MYC signaling that are known as nutrient-sensing pathways were more active in pre-natal HSCs, such as YS-MPs, AGM-HSCs and FL-HSCs (Fig. 2C), presumabely related to their rapid proliferation at early developmental stages. In contrast, the LKB-AMPK pathway was inactive in YS-MPs and AGM-HSCs while highly active in UCB- and PB-HSCs (Fig. 2D). AMPK signaling is known as a master regulator to restrict cell growth under poor conditions and might play important roles in these HSCs to maintain their quiescency. Notably, both PI3K-AKT-mTOR and AMPK signaling were active in FL-HSCs (Fig. 2D), indicating these two pathways were balanced to maintain the rapid self-renewal in FL-HSCs. Autophagy was shown to be critical to maintain HSC normal functions (Mortensen et al. 2011; Warr et al. 2013; Ho et al. 2017), but only showed high expression in PB-HSPCs (Fig. 2E). FOXO pathway that was known to promote detoxication of ROS was more active in UCB- and PB-HSPCs, but not YS-MPs, AGM-HSCs and FL-HSCs (Fig. 2E). In all, these data indicate that differential metabolism pathways were employed in human primary HSPCs for the differential hematopoiesis requirement at various human developmental stages.

### Differential lineage potentialities in human primary HSPCs over development

**Fig. 3:**
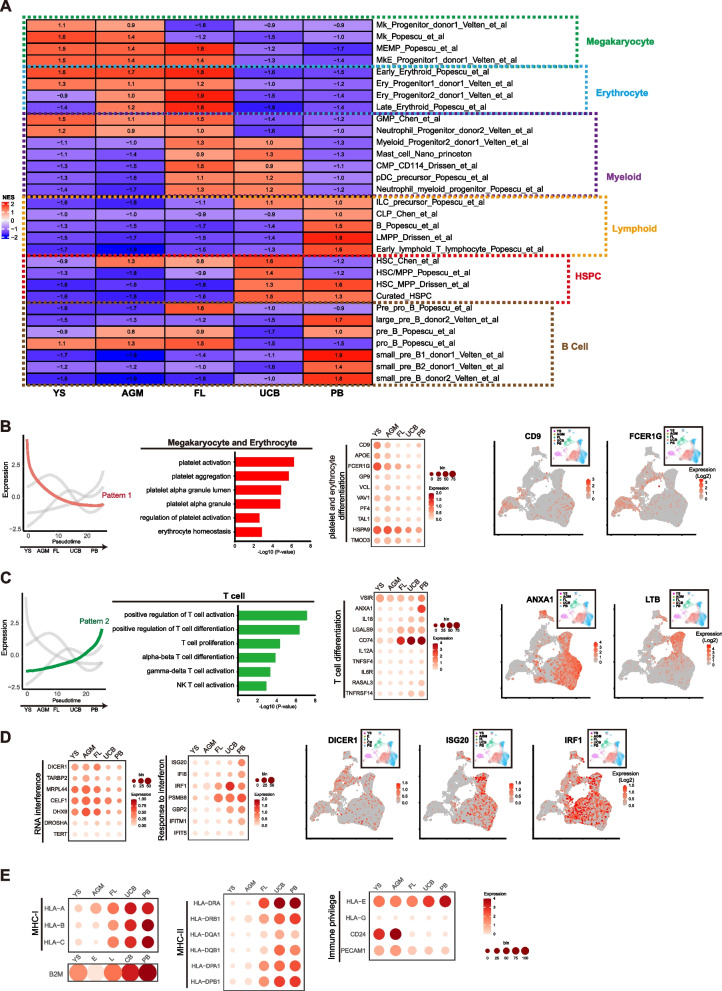
Differential lineage potentialities in HSPCs from different developmental stages. **A** Heatmap of GSEA score in different HSPCs for the indicated HSC lineage gene sets selected from published papers. GSEA scores indicated by NES number are shown and differentially colored. **B-C** Lineage potentiality genes display differential dynamics in HSCs across pseudotime developmental path. Megakaryocyte and erythrocyte potentiality genes are down-regulated (**B)** while the T cell potentiality genes are up-regulated **(C)**. Bar plots indicate the enrichment of selected GO terms; dot plots indicate the expression level of selected genes involved in the biological process as shown on the left; feature plots show the expressions of selected genes in different HSCs. Color indicate expression level (TPM, Log-scaled).** D** Expressions of RNAi and interferon genes in different HSCs. Color indicate expression level (TPM, Log-scaled).** E** Expressions of MHC and selected immune privilege genes in different HSCs. Color indicates the scaled mean expression level (TPM, Log-scaled) and the size of the dot indicates the percentage of cells expressing the given gene

Numerous studies have revealed that the lineage composition in hematopoietic compartment varied a lot at different developmental stages and sites over human development (Copley and Eaves 2013; Popescu et al. 2019; Roy et al. 2021). For example, erythroid/megakaryocytes (Er/Mk) lineages were predominate in human early fetal liver while lymphoid lineages became evident in fetal bone marrow (FBM) (Popescu et al. 2019; Roy et al. 2021). We sought to examine whether these developmental changes were manifested in primary HSPCs. We performed GSEA on LP-HSPCs based on the published human hematopoietic gene sets (Fig. 3A). YS-MPs and AGM-HSCs displayed dominant Er/Mk but less lymphoid potentiality, particular lacked B cell potency (Fig. 3A). Myeloid lineage (granulocyte-monocyte, GM) potency was mostly manifested in FL-HSCs and UCB-HSCs (Fig. 3A), consistent to previous studies in mouse model (Copley and Eaves 2013; Bowie et al. 2007). Lymphoid potentialities, represented by B cell and early T cell profile were mostly evident in PB-HSCs (Fig. 3A). Notably, HSC signature profile was mostly evident in UCB-HSCs (Fig. 3A), indicating the developmental maturity of HSCs at birth stage. Furthermore, Er/Mk potentiality profile was dynamically down-regulated while lymphoid potential was up-regulated in human LP-HSPCs over development (Fig. 3B-C).

Interestingly, RNA interference (RNAi) pathway was much more active in pre-natal HSPCs, but substantially downregulated in UCB- and PB-HSCs (Fig. 3D). Conversely, the interferon response pathway was inactive in YS-MPs and AGM-HSCs, but became active in FL-HSCs and peaked in PB-HSCs (Fig. 3D). Since RNAi and interferon pathway represent innate and adaptive immunity against virus infection respectively, these data indicate that differential virus-defense pathways are utilized in human HSCs at different developmental stages. Lastly, the major histocompatibility complex (MHC) genes were inactive in YS-MPs and AGM-HSC and started to express in FL-HSCs (Fig. 3E). To the contrary, the immune privilege genes were highly expressed in YS-MPs and AGM-HSCs (Fig. 3E). Together, these data show that human primary HSPCs have intrinsically differential lineage potentialties in ontogeny.

### Gene regulatory networks (GRNs) underlie differential properties in human primary HSPCs over development

**Fig. 4:**
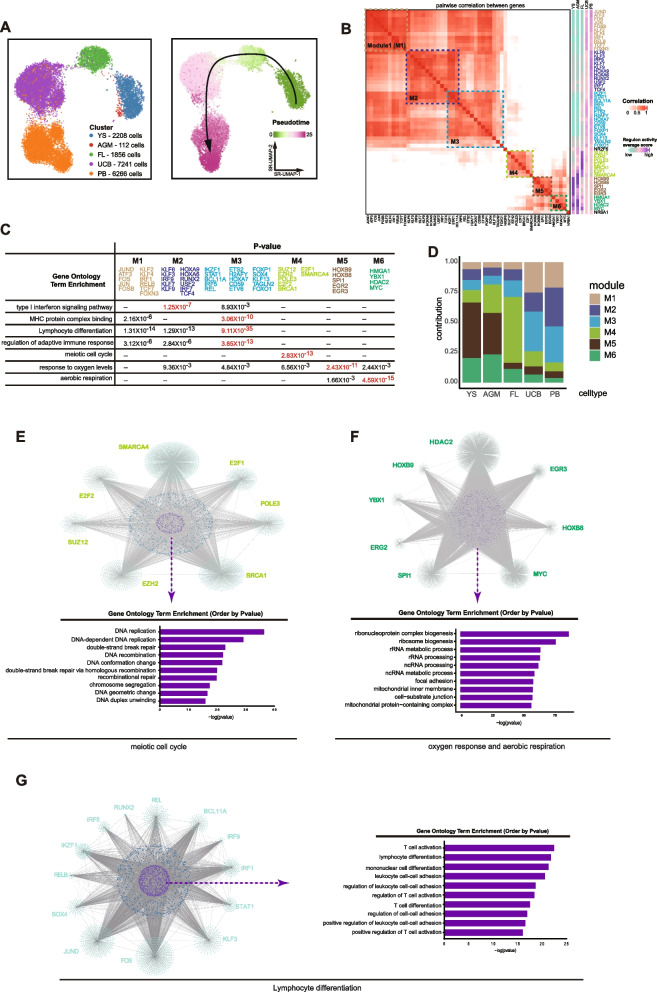
Gene regulatory networks (GRNs) underlie differential properties in human HSPCs. **A** UMAP plot based on the regulon activity score colored by samples (left) or pseudotime developmental path (right). **B** Pairwise correlation plot of regulons in different HSPCs. Regulon activity score and co-relation score are indicated by different colors. 6 highly co-related regulon modules are highlighted. **C** GO enrichment analyses on 6 co-related regulon modules. The most enriched GO terms in each module are shown. The *p*-values for each GO term smaller than $1\times {10}^{-3}$ are shown and highlighted for those smaller than $1\times {10}^{-7}$. **D** Distribution of six regulon modules in different cell types. **E–G** Regulatory networks by indicated regulon modules. The blue dots indicate genes which are shared targets of two regulators and the purple dots indicate genes which are shared targets of more than two regulators. GO analysis is performed on target genes shared at least by two regulators in each module

We then investigated molecular regulators that control the differential properties of human HSPCs over development. We performed single-cell regulatory network inference and clustering (SCENIC) to generate GRNs represented by regulon containing a set of genes co-expressed and regulated by transcription factors (TFs) (Aibar et al. 2017). Regulon activities inferred from human HSPCs formed highly related clusters consistent to their developmental stages but displayed a dynamic pseduotime developmental path following the development timing (Fig. 4A). To identify regulons that control the particular HSC property in HSC ontogeny, we generated highly co-related regulon modules across HSPCs at different stages (Fig. 4B). We detected 6 highly co-related GRN modules that control different key properties in human HSCs at different developmental stages (Fig. 4B-C). For example, module 3 regulons (M3) mainly regulates lymphoid potentiality and adaptive immune response, while M4 controls cell cycle and M5/M6 regulate aerobic respiration (Fig. 4C). HSPCs at different stages showed differential activities of these regulon modules (Fig. 4D). FL-HSCs that have the highest self-renewal showed extremally high M4 activity (Fig. 4D). M4 contains 7 regulators and some of them had been shown to regulate HSC self-renewal, such as EHZ2, SUZ12 etc. (Xie et al. 2014; Kamminga et al. 2006; Lee et al. 2015). The gene network co-regulated by the 7 regulators in M4 was high enriched in functions related to DNA replication and repair (Fig. 4E). Early stage HSPCs such as YS-MPs and AGM-HSCs showed substantially higher activity of M5/M6 modules that mainly functions to control oxygen metabolism (Fig. 4C-D),which might be consistent to their highly active metabolism pathways (Fig. 2). Indeed, the gene set co-regulated by M5/M6 regulators was highly enriched in genes related to ribosome, mitochondrial synthesis etc. (Fig. 4F). UCB- and PB-HSPCs showed the highest regulon activity in M1/M2/M3 modules that mainly control the lymphoid potentiality and immune functions (Fig. 4C-D). The gene set co-regulated by TFs from M1-3 modules was extremely enriched in functions related to lymphoid potentiality (Fig. 4G), indicating that the M1-3 contain the intrinsic determinants of lymphoid potentiality in human HSCs. Together, these data suggest that the intrinsic GRNs controlling differential properties of human primary HSPCs undergo dynamic changes in human HSC ontogeny.

### Human iPSCs derived HSPCs resemble early embryonic stage HSPCs

**Fig. 5:**
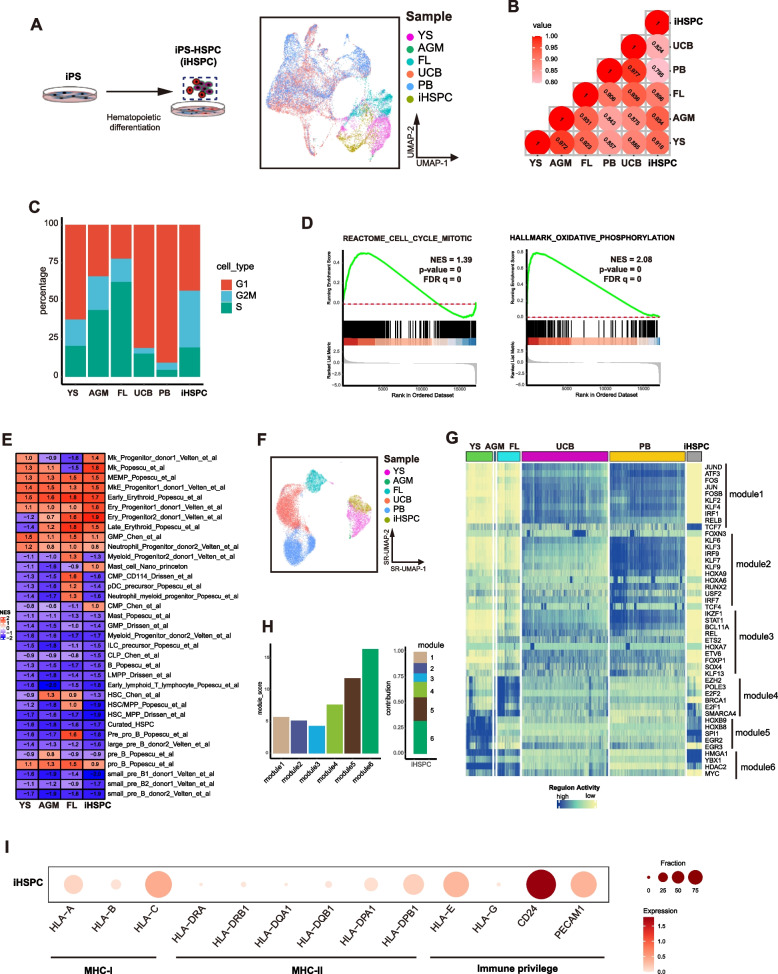
Human iPSCs derived HSPCs are close to early embryonic stage HSPCs. **A** UMAP plot of the single cell transcriptomes of human iPSCs derived HSPCs (iHSPCs) and other indicated human HSPCs. **B** Pearson ‘s correlation plot of indicated HSPCs. Co-relation value scores are indicated. **C** Predicated cell cycle stages indicated HSPCs. **D** GSEA score plots of the gene sets with cell cycle or oxidative phosphorylation in iHSPCs.** E** Heatmap of GSEA score in indicated HSPCs for the indicated HSC lineage gene sets selected from published papers. GSEA scores indicated by NES number are shown and differentially colored.. **F** UMAP plot based on the regulon activity score colored by samples. **G** Heatmap of scaled regulon activity of indicated HSPCs. Regulators shown in the plot are selected from Fig. 4B. **H** Distribution of regulon modules in iHSPCs. **I** Expression of indicated genes in iHSPCs. Color indicates the scaled mean expression level (TPM, Log-scaled) and the size of the dot indicates the percentage of cells expressing the given gene

We previously reported a monolayer and defined condition to differentiate human iPSCs into HPSCs with multi-lineage potency (Zhang et al. 2019). Human iPSCs derived HSPCs (iHSPCs) generated based on this protocol could give rise to various blood/immune cells including Er/Mk, myeloid cells and immune cells such as NK, B and T cells both in vitro and in vivo (Zhu et al. 2020). Single cell transcriptome data showed that these iHSPCs clustered together with the early stage HSPCs and were close to the AGM-HSCs (Fig. 5A-B). iHSPCs also showed more active cell cycle prediction and highly active OXPHOS state (Fig. 5C-D). Resembling YS-MP and AGM-HSCs, iHSPCs displayed higher Er/MK but lower Myeloid/lymphoid potentialities (Fig. 5E). GRNs from iHSPCs formed an indiscrete cluster with YS-MPs and AGM-HSCs, but separated from FL-, UCB- and PB-HSCs (Fig. 5F). Regulon activities of M1-3 that control lymphoid potentiality were lower in iHSPCs compared with other regulon activities (Fig. 5G-H). Like YS-MPs and AGM-HSCs, M5/6 regulons that control metabolism were highly active in iHSPCs (Fig. 5G-H). M4 regulons that control HSC self-renwal was active in iHSPCs, but lower than that in FL-HSCs (Fig. 5G-H). In addition, similar to YS-MPs and AGM-HSCs, iHSPCs showed much lower expression of MHC genes but higher immune privilege gene expressions (Fig. 5I). All these data indicate that human iPSCs derived HSPCs resemble much to the early embryonic stage HSPCs with preferential manifestation of Er/Mr potentialities while lower lymphoid potentiality. These findings are consistent to the functional analysis that iHSPCs gave rise into more Er/Mr but less lymphoid lineage output in differentiation (Zhu et al. 2020; Dou et al. 2016; Uenishi et al. 2018).

### Promoting iHSPC functions by key TFs

**Fig. 6:**
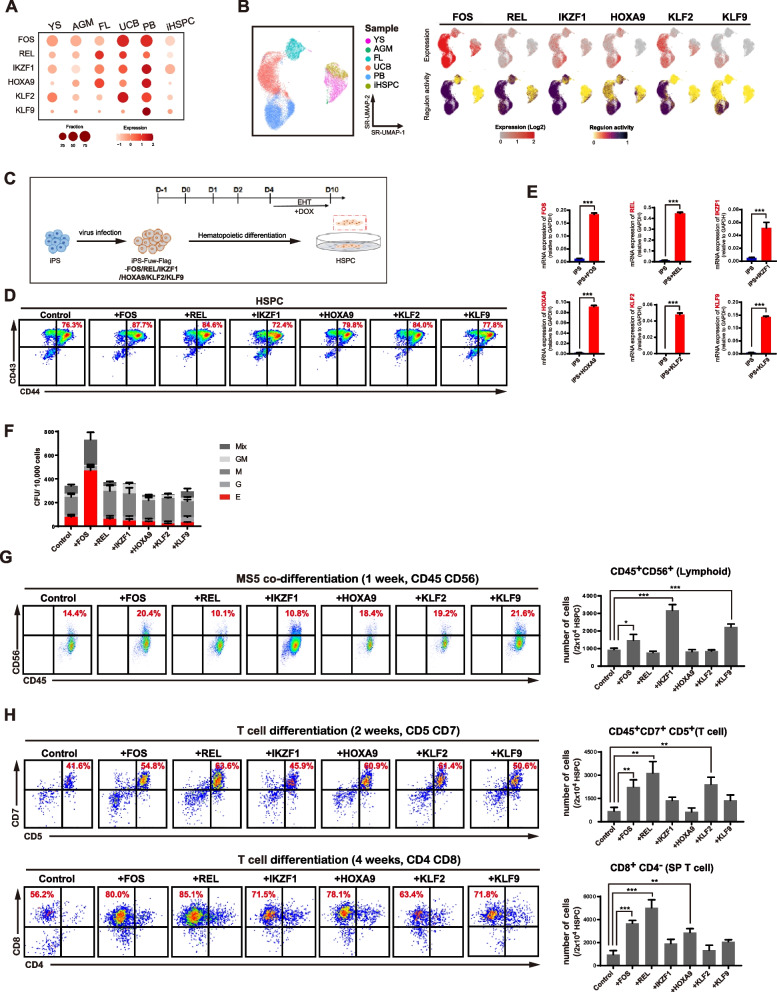
Expression of key TFs promotes iHSPC functions. **A** Expression of indicated genes in different HSPCs. Color indicates the scaled mean expression level (TPM, Log-scaled) and the size of the dot indicates the percentage of cells expressing the given gene.** B** Regulon activity and expressions of indicated regulators.** C** Scheme of the experiment design.** D** FACS analysis of the indicated iHSPCs with expression of the indicated TFs. **E** Expression of indicated TFs in each indicated iHSPCs by qRT-PCR assay.** F** CFU analysis on indicated iHSPCs with expression of key TFs.** G** Analysis the NK differentiation on indicated iHSPCs. iHSPCs are co-cultured with MS5 cells and NK cells are analyzed by FACS based on CD45^+^CD56^+^. The number of NK cell generated per seeded 2X10^4^ HSPCs are shown. **H** Analysis the T differentiation on indicated iHSPCs. iHSPCs are co-cultured with OP9-DL4 cells and T progenitor cells are analyzed by FACS based on CD45^+^CD7^+^ or CD5^+^ at 2 weeks differentiation. T mature CD8^+^CD4^−^ cells are analyzed at 4 weeks differentiation. The number of indicated T progenitor or mature T cells generated per seeded 2X10^4^ HSPCs are shown. The significance level was determined by unpaired two-tailed Student’s t tests. *, *p* < 0.05; **, *p* < 0.01; ***, *p* < 0.001. The data represent the mean + SEM from three independent repeats (*n* = 3). All error bars throughout the figure represent the SEM from three independent replicates (*n* = 3)

Since iHSPCs display less lymphoid potentiality, we have interests to investigate whether introducing the identified TFs could promote iHSPC functions. Six TFs in M1-3 that showed higher expression and regulon activities in UCB- and PB HSPCs were selected (Fig. 6A-B). These TFs were expressed at lower level in iHSPCs (Fig. 6A-B). We firstly generated an inducible approach to express these TFs in hiPSCs by doxycycline (DOX) treatment (Supplemental Fig. 1). We then differentiated these cells into iHSPCs based on previously published protocol and trigged expression of these TFs at endothelia-hematopoietic transition (EHT) stage (Fig. 6C-D). The generated iHSPCs expressed typical HPC markers such as CD43/CD44 (Zhu et al. 2020) and high level of the transduced TFs (Fig. 6E). Upon further differentiation by blood colony-forming unit (CFU) assay, FOS expressed iHSPCs showed substantially enhanced CFU forming compared with control and other iHSPCs (Fig. 6F). Importantly, the mixed cell type CFUs that indicate the HPCs with multi-lineage potential were dramatically enhanced in FOS expressed iHSPCs (Fig. 6F). Based on another differentiation approach, MS5-cocluture, iHSPCs exressed FOS, IKZF1 or KLF9 showed substantially enhanced NK cell generation (Fig. 6G). To examine T cell potential, we differentiated these iHSPCs on OP9-DL1 that was reported to support T cell generation *in vitro* (Awong et al. 2009). At 2 weeks differentiation, FOS, REL and KLF2 showed more dramatic effect to promote the generation of CD45 + /CD7 + human T progenitors (Fig. 6H). At 4 weeks differentiation for T cell maturation, FOS, REL and HOXA9 showed much more effects in generation of CD8^+^/CD4^−^ single positive T cells (Fig. 6H). Together, these data indicate that the functions of HSPCs derived from iPSCs could be enhabced by key TFs that control the intrinsic properties in HSPCs.

## Discussion

Human hematopoiesis begins at yolk sac being the first wave to generate blood cells for the immediate needs of growing embryo and undergoes site- and stage-specific changes in its ontogeny (Laurenti and Göttgens 2018; Dzierzak and Bigas 2018; Ivanovs et al. 2017; Copley and Eaves 2013; Popescu et al. 2019; Mack et al. 2021). The cellular architecture and lineage composition varies in hematopoietic compartment at different developmental stages and sites (Haas et al. 2018; Copley and Eaves 2013; Popescu et al. 2019; Calvanese et al. 2022; Ranzoni et al. 2021; Roy et al. 2021). Residing on the top of hematopoietic hierarchy, the primary HSPCs also undergo dynamic changes in their key properties, such as cell cycling, lineage potentiality etc. (Haas et al. 2018; Copley and Eaves 2013; Roy et al. 2021; Mack et al. 2021). In this study, we define the dynamic property changes and the underlying GRNs in primary HSPCs across various human developmental stages and sites. Firstly, human primary HSPCs display differential metabolic activities and regulatory pathways over development. HSPCs at early embryonic stages such as YS-MPs, AGM-HSCs display much more active metabolism, such as higher level of glucose metabolic activities. In contrast, later stage HSPCs, the UCB- and PB-HSPCs show much reduced metabolism. Consistent to the differential metabolic activities, YS-MPs and AGM-HSC are highly proliferative while UCB- and PB-HSPCs are mostly quiescent. The PI3K-AKT-mTOR signaling is more active at YS-MPs and AGM-HSCs while AMPK signaling is active at UCB- and PB-HSPCs. Interestingly, FL-HSCs display an intermediate activity on metabolism and both PI3K-AKT-mTOR and AMPK signaling. Secondly, human primary HSPCs show dynamic changes in linage potentialities in ontogeny. Early embryonic stage HSPCs displayed dominant Er/Mk but less lymphoid potentiality, particular the B cell potency. Myeloid lineage (granulocyte-monocyte, GM) potency was mostly manifested in FL-HSCs and UCB-HSCs (Fig. 4A). Lymphoid potentialities, represented by B and early T cell profile progressively get acquired over development and are mostly evident in PB-HSCs. These data indicate that the differential lineage composition of hematopoietic compartment over development has been intrinsically manifested in primary HSPCs. HSPCs derived from human iPSCs (iHSPCs) via the strategy that repopulates main stages of early hematopoietic development mostly resemble the early stage HSPCs, particularly the AGM-HSCs (Fig. 5). Interestingly, early embryonic stage HSPCs display low expression of MHC genes but much higher immune privilege genes, which might promote their immune escape from maternal immune system at early days post-conception. Consistently, iHSPCs also express much lower MHC genes and higher immune privilege genes, which is advantageous to serve as the universal cell line for potential applications.

More importantly, we generate GRNs underlie the differential properties of naïve HSPCs over development. Six highly co-related GRN modules in various stage specific HSPCs were identified and these modules control different key HSC properties. Particularly, module 4 (M4) is specifically active in FL-HSCs that are known to undergo rapid self-renewal in vivo. Consistently, the major function of GRN controlled by M4 is highly enriched in regulation of self-renewal, such as DNA replication, chromosome segregation etc. Indeed, couple TFs in M4 have been shown to promote self-renewal of HSCs in various model systems, such as EZH2 and E2F1 (Xie et al. 2014; Kamminga et al. 2006; Matsumoto and Nakayama 2013; Gala et al. 2001). Another important HSC property is the lymphoid potentiality as malfunction of lymphoid lineage differentiation is highly related to various diseases, such as cancer. We identified highly co-related modules(M1-3) that are highly active in UCB- and PB-HSPCs and regulate lymphoid and immune function. Several TFs in this module have been shown to promote HSC function or lymphoid lineage specification such as FOS, REL, KLF2 and HOXA9 (Yaseen et al. 1994; Fallahi et al. 2017; Hart et al. 2012; Ramos-Mejía et al. 2014). These data indicated the developmental changes and key properties in HSC ontogeny are being intrinsically controlled by the specific GRNs and key TFs.

In recently years, hematopoietic differentiation of human PSCs have been described by different groups (Dou et al. 2016; Wahlster and Daley 2016; Ditadi et al. 2017). However, induced HSPCs (iHSPCs) derived from human PSCs are far from fully functional. The self-renewal and long-term engraftment of iHSPCs in vivo have not been described. Also, the lineage output of iHSPCs is very limited, particularly lack the lymphoid potential that is typically known as the marker for definitive hematopoiesis. We previously reported a defined, mono-layer approach to generate iHSPCs that have multi-lineage potential to give rise to various myeloid cells and immune cells, but the myeloid lineage output is dominant (Zhu et al. 2020). Our analysis here show that these iHSPCs resemble most close to the early embryonic stage HSPCs, such as YS-MP and AGM-HSCs, which is understandable as PSC differentiation in vitro recapitulates early embryonic development and lacks embryonic niche for HSC maturation, such as fetal liver. GRNs and major TFs underling key HSC functions identified here might help to promote the function of iHSPCs generated in vitro. Indeed, introducing the selected TFs control lymphoid potentiality promotes lymphoid lineage output in iHSPCs in differentiation in vitro. However, we failed to detected the contribution of iHSPCs derived T cells in NSG mice (data not shown). NSG mice are highly immunodeficient and might lack conditions for T cell maturation, such as functional thymus. We also haven’t examined other identified TFs one by one in human HSC functions,such as metabolism factors, self-renew factors etc. Nevertheless, GRNs and their TFs underlying key properties of human HSCs provide a valuable guide to promote the full function of HSPCs from human PSCs.

## Methods

### Cells culture and maintenance

hPSCs were maintained on Matrigel (1:100 dilution; BD) coated plates in mTeSR1 medium (Stem Cell Technologies) supplemented with 1% penicillin–streptomycin (Hyclone). Medium was changed every day and cells were passaged 1:3 onto fresh Matrigel coated plates every 3 days using 0.5 mM ethylenediaminetetraacetic acid disodium salt (EDTA-2Na). All of the hPSCs cell lines mentioned above were cultured under 37 °C, 20% O_2_ and 5% CO_2_ condition and had been tested to be free of mycoplasma contamination. The experiments using human cell materials have been approved by the Ethics Committee at Guangzhou Institutes of Biomedicine and Health, Chinese Academy of Sciences.

### Generation of regulators forced-expression hPSCs

The human regulators gene were cloned into a lentiviral vector tetO-FUW for tet-inducible expression. Lentivirus was produced in 293 T cells by cotransfecting the tetO-FUW-regulators with three helper plasmids (pRSV-REV, pMDLg/pRRE, and vesicular stomatitis virus G protein expression vector), which provide the essential elements to package lentivirus. Viral supernatants were collected at 48 h after transfection and passed through a 0.45 μm filter to remove cell debris, then subjected to ultracentrifugation (20,000 × g for 3 h at 4 °C). hPSCs were transduced with lentivirus. The expression of regulators are induced by exogenous addition of doxycycline (DOX) (2 μg per mL) and the positive cells were selected by puromycin (1 μg per mL).

### Generation of regulators knock-down HSPC

Fresh mobilized PB samples were obtained as a gift from Department of Hematology, The Third Affiliated Hospital, Sun Yat-sen University. They were processed within 24 h after received. Mononuclear cells were isolated firstly by removing of red blood cells using ACK lysis buffer, and then CD34 + fraction was separated by CD34 Microbead kit and magnetic-activated cell-sorting separation columns (Miltenyi Biotec) according to the manufacturer’s instruction. The shRNA sequences targeting against regulators were designed from Sigma official website, then these sequences were connect into backbone pLKO.1. Lentivirus particles were produced by co-transfecting 293 T cells. 5 × 105 PB CD34 + cells were pre-stimulated for 24 h (cells were culture at ultra-low attachment 24 well plate with SCGM (CellGenix)), then 20ul lentivirus particles were added into the medium and maintained for 36 h. Transduced cells were washed and cultured with fresh medium.

### Western blot assays

Cells were lysed on ice with 200 μL of RIPA buffer (Beyotime) for 15 min and separated by 12% sodium dodecyl sulfate–polyacrylamide gel electrophoresis (SDS-PAGE) before being transferred onto polyvinylidene difluoride (PVDF) membranes (Millipore). The membranes were blocked in 5% nonfat milk for 1 h and incubated overnight at 4 °C with the appropriate diluted primary antibodies or anti-flag/GAPDH antibody. Subsequently, the membranes were incubated with HRP-conjugated secondary antibody for 2 h at room temperature and HRP was detected by ECL (Advanste) and visualized by SmatChemi Image Analysis System (SAGECREATION).

### Hematopoietic differentiation of hPSCs

Prior to differentiation, the hPSCs should be 80% ~ 90% confluent and were dissociated into single cells using Accutase (Sigma). And then cells were plated onto Growth Factor Reduced (GFR) Matrigel (1:100 dilution; BD) coated six-well plates at a proper initial density about 3 × 10^5^/well. Especially, in order to inhibit hPSCs apoptosis, thiazovivin (0.1 μM, Selleck) was added in the medium. After 24-36 h culture, the cells were about 10% confluent and this day was designated as day 0 (D0). Then, the hPSCs were induced for stepwise differentiation in basal medium (BM) supplemented with cytokines and inhibitors with the following days. D0-D1: 40 ng/ml BMP4 (Peprotech), 30 ng/ml ACTIVIN A (Sino Biological), 20 ng/ml bFGF (Sino Biological), 6 μM CHIR99021 (Selleck) and 10 μM LY294002 (Selleck); D1-D2: 30 ng/ml BMP4, 1 μM A8301 (Selleck) and 2 μM IWR-1-endo (Selleck); D2-D4: 40 ng/ml VEGF (Sino Biological) and 50 ng/ml bFGF; D4-D8: 40 ng/ml VEGF, 50 ng/ml bFGF, 10 μM SB431542 (Selleck), 10 ng/ml SCF (Peprotech), 50 ng/ml TPO (Sino Biological), 10 ng/ml IL3 (Sino Biological), 50 ng/ml IL6 (Sino Biological) and 50 ng/ml FLT3L (Peprotech). BM: DMEM/F12 (GIBCO) + 1% penicillin–streptomycin (Hyclone) + 1% insulin-transferrin-selenium (ITS, GIBCO) + 70 μg/ml vitamin C (Vc, 2-Phospho-L-ascorbic acid trisodium salt solution, Sigma). Particularly, the osmotic pressure of the HDM was adjusted by 9% NaCl to about 340. The hematopoietic differentiation medium in each step should be changed every day and the differentiating cells were differentiated in 37 °C, 20% O_2_ and 5% CO_2_ condition. The expression of regulators are induced by exogenous addition of doxycycline (DOX) (2 μg per mL) from D4.

### Flow cytometry

Single cell suspension was prepared and filtered through 70 μm filter. Then, cells were stained by multicolor antibody combinations in DPBS supplemented with 2% FBS and incubated in 4 °C for 20–30 min. The cells were detected by flow cytometry. Antibodies were listed in Supplementary Table 1.

### Quantitative real-time PCR (qRT-PCR)

The total RNA were extracted from cells using the RaPure Total RNA Micro Kit (Magen) and two μg RNA were reversely transcribed into cDNA with a HiScript II 1st Strand cDNA Synthesis Kit (Vazyme). Then, qRT-PCR was performed with ChamQ SYBR qPCR Master Mix (Vazyme) and a CFX96 machine (Bio-Rad). GAPDH were used for normalization. All data were analyzed with 3 replicates and all primers used in this study were listed in the Supplemental Table 2.

### CFU assay

The CFU assay was performed according to the manufacturer’s instruction of Methocult H4435 (Stem Cell Technologies). Firstly, an indicated number of single cells were suspended into 120 μl IMDM medium supplemented with 2% FBS (Biological Industries), and then add the cell suspension to 1 ml Methocult H4435. Next, transferred the mixture to 35 mm ultra-low attachment plates (Stem Cell Technologies) and rotated gently to spread methylcellulose medium over the surface of the dish. Placed 3 dishes within a 100 mm petri dish containing with 3 mL sterile water and incubated the dishes in 37 °C, 20% O_2_ and 5% CO_2_ condition. The CFUs were classified and calculated according to the morphology after 2 weeks. All data were analyzed with 3 replicates.

### lympho-myeloid lineage differentiation

MS-5 cells were seeded onto 0.1% gelatin coated 24-well plate at a initiating density of 2 × 10^4^/well in α-MEM medium (Thermo Fisher) supplemented with 10% FBS (Gibco), 1% penicillin–streptomycin (Hyclone) and 1% GlutaMAXTM (Gibco). 24 h after plating of MS-5 stroma, 1 × 104 cells were added into each well in the presence of 0.1 μM DuP-697 (Biovision), 20 ng/ml SCF (Peprotech), 10 ng/ml G-CSF (Peprotech), 10 ng/ml FLT3L (Peprotech), 10 ng/ml IL-2 (Peprotech) and 10 ng/ml IL-15 (Sino Biological). Half of the medium was changed twice every week and cocultures were transferred onto fresh MS-5 stroma every two weeks through 40 μm filter to remove the stromal cells. All of the cells in each well were harvested and analyzed by flow cytometry at week 1. Antibodies were listed in Supplementary Table 1.

### T cell differentiation

OP9-hDLL4 cells were seeded onto 24-well plate coated by 0.1% gelatin at a density of 2 × 10^4^/well in α-MEM medium (Thermo Fisher) supplemented with 20% FBS (Gibco), 1% penicillin–streptomycin (Hyclone) and 1% GlutaMAX™ (Gibco). One day after plating of OP9-hDL4 cells, 1 × 10^4^ cells were deposited into each well containing 10 ng/ml SCF (Peprotech), 5 ng/ml FLT3L (Peprotech) and 5 ng/ml IL-7 (Sino Biological). Half of the medium was changed twice every week. Change the medium to α-MEM medium (Thermo Fisher) supplemented with 15% FBS (Gibco), 1% penicillin–streptomycin (Hyclone), 1% GlutaMAX™ (Gibco), 500 ng/ml anti-CD3 monoclonal antibody (OKT3), 10 ng/ml IL-2 (Peprotech), 10 ng/ml IL-7 (Sino Biological) and 10 nM dexamethasone from week 3 (day 21). After three days, OKT3 was removed and the culture continued week 4 (day 28). Harvested cells were analyzed by flow cytometry at week 2 and week 4. Antibodies were listed in Supplementary Table 1.

### Single cell RNA-seq data preprocessing

10X sequencing data were processed with Cellranger (version 6.1.2) with default parameters based on the human reference genome GRCh38. Digital gene expression matrices were imported into R (version 4.0.2) for downstream analyses using the Seurat package (version 4.1.1). Quality control was performed by only retaining cells with UMI counts between mean ± 2*SD* of total mRNA and less than 20% mitochondrial expression for each sample. Only genes expressed in at least 3 cells and encoded proteins were included.

### Data integration

To remove batch effect among different datasets, Seurat (version 4.1.1) was used to integrate data. The Seurat object of each sample were normalized using the NormalizeData function. FindVariableFeatures function was performed to find top 3000 highly variable genes. SelectIntegrationFeatures function was used to select features for downstream integration and PCA (principle component analysis) was run on each object based on scaled data using integration features. FindIntegrationAnchors and IntegrateData functions were applied according to the reference-based integrate workflow to integrate all samples.

### Cell cycle analysis

To identify cell cycle phases of cells, CellCycleScoring function was performed to score and predict based on the expression of cell cycle-related genes provided by Seurat.

### Dimension reduction

For visualization, the integrated data was scaled using ScaleData function with the parameter "vars.to.regress" = "Phase". After performing RunPCA, the integrated data was projected into Uniform Manifold Approximation and Projection (UMAP) dimensional reduction based on 30 principal components using RunUMAP function.

### DEGs identification

FindMarkers function was performed to identify the top DEGs among different sources of HSPCs. The correlationship between DEGs was then calculated using cor function and was visualized using Heatmap function from ComplexHeatmap (version 2.6.2).

### Pseudo-time trajectory analysis

Pseudo-time trajectory was constructed with Monocle2 (version 2.10.1) following the official vignette. Highly variable genes were identified as genes with more than 1.5 times of fitted dispersion evaluated using dispersionTable function, and reduceDimension function was performed with the Discriminative Dimensionality Reduction with Trees (DDRTree) method and orderCells function followed. Significant DEGs along pseudo-time were identified and classified into different patterns by differentialGeneTest function.

### Functional enrichment analysis

Gene ontology enrichment was performed using enrichGO function from clusterProfiler (version 3.18.1) package. Gene set enrichment analysis was performed using GSEA function from clusterProlier.

### SCENIC analysis and construction of regulatory network

To investigate gene regulatory network among HSPCs from different sourses, pySCENIC (version 0.11.0) was used to identify the core TFs and their functional targets. Regulons with variable activity among different samples were selected to calculate the correlationship and classified into different functional module based on functional enrichment. The regulatory network of different modules was visualized by Cytoscape (version 3.9.1) using yFiles Organic layout and Compound Spring Embedder (COSE) layout.

### Statistical analysis

Data were presented as mean + SEM, and statistics were determined by unpaired two-tailed Student’s test (t-test). P value < 0.05 were considered statistically significant. *, *p* < 0.05; **, *p* < 0.01; ***, *p* < 0.001. No statistical Method was used to pre-determine sample size. No samples were excluded for any analysis. No randomization was used for allocating animal group. No blinding done in animal experiments.

### Supplementary Information

**Additional file 1: Supplemental Fig. 1. **A: Morphology of key TFs-modified iPSCs. B: Western blot assays confirming the over-expression of key TFs in indicated iPSCs.C: Morphology of indicated iHSPCs.

**Additional file 2: Supplementary Table 1.** Antibodies.

**Additional file 3: Supplementary Table 2.** qRT-PCR primers.

## Data Availability

The scRNA-seq data generated during the current study has been deposited in the NCBI Gene Expression Omnibus under ID code GSE224972.

## Abbreviations

HSPCs: Hematopoietic stem/progenitor cells
HSCs: Hematopoietic stem cells
HPCs: Hematopoietic progenitor cells
YS: Yolk sac
AGM: Aorta-gonad-mesonephros
FL: Fetal liver
UCB: Umbilical cord blood
PB: Peripheral blood
GRNs: Gene regulatory networks
EMPs: Erythromyeloid progenitors
NK cells: Natural killer cells
Er: Erythroid
Mk: Megakaryocyte
Ly: Lymphoid
My: Myeloid
MPs: Multipotential progenitors
hPSCs: Human pluripotent stem cells
hiPSCs: Human induced pluripotent stem cells
LP: Less-lineage primed
MHC: Major histocompatibility complex
GSEA: Gene set enrichment analysis
OXPHOS: Oxidative phosphorylation
ROS: Reactive oxygen species
FBM: Fetal bone marrow
BM: Bone marrow
RNAi: RNA interference
SCENIC: Single-cell regulatory network inference and clustering
TFs: Transcription factors
M1/2/3/4/5/6: Module 1/2/3/4/5/6
iHSPCs: Human iPSCs derived HSPCs
EHT: Endothelia-hematopoietic transition
CFU: Colony-forming unit
GM: Granulocyte-monocyte
