# Supplementary figures and images for: Characterization of gene regulatory networks underlying key properties in human hematopoietic stem cell ontogeny
Source: Cell Regen. 2024 Apr 17;13:9. doi: 10.1186/s13619-024-00192-z (PMC11024070; doi:10.1186/s13619-024-00192-z)

Supplemental figure 1

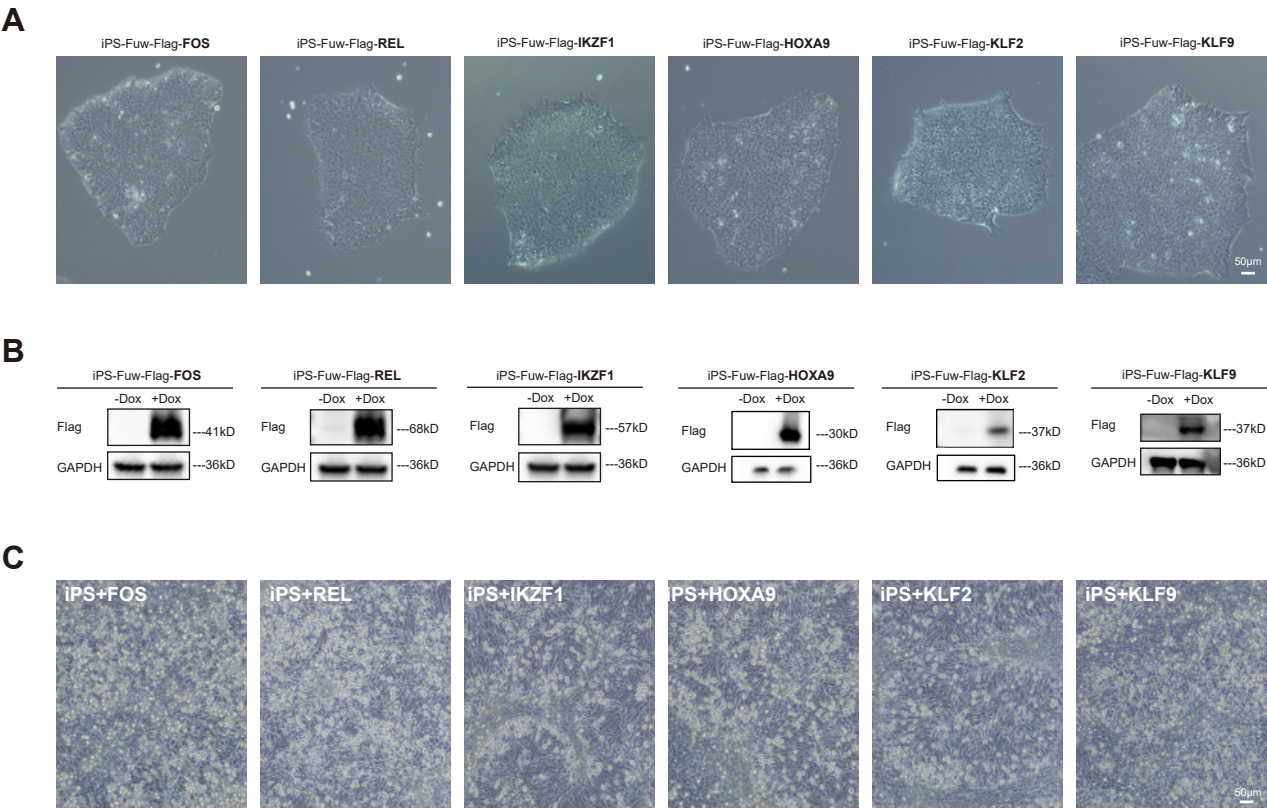

Supplement: Supplementary file 1 — Additional file 1: Supplemental Fig. 1. A: Morphology of key TFs-modified iPSCs. B: Western blot assays confirming the over-expression of key TFs in indicated iPSCs.C: Morphology of indicated iHSPCs. [file 13619_2024_192_MOESM1_ESM.pdf]
