# Supplementary material for: Characterization of gene regulatory networks underlying key properties in human hematopoietic stem cell ontogeny
Source: Cell Regen. 2024 Apr 17;13:9. doi: 10.1186/s13619-024-00192-z (PMC11024070; doi:10.1186/s13619-024-00192-z)
Supplement: Supplementary file 2 — Additional file 2: Supplementary Table 1. Antibodies. [file 13619_2024_192_MOESM2_ESM.pdf]

**Supplemental Table 1 Antibodies**

| Antibodies | Vendor | Cat# | Dilution |
|---|---|---|---|
| Anti-human CD43-APC | BD Biosciences | 560198 | 1:100 |
| Anti-human CD44-FITC | BD Biosciences | 555478 | 1:100 |
| anti-human CD45-PE | eBioScience | 12-9459-42 | 1:100 |
| anti-human CD56-PerCP-Cy5.5 | Biolegend | 318322 | 1:100 |
| anti-human CD45-APC | BD Biosciences | 560973 | 1:100 |
| anti-human CD45-PE-Cy7 | Biolegend | 304016 | 1:100 |
| anti-human CD33-PE | BD Biosciences | 347787 | 1:100 |
| anti-human CD56-APC | BD Biosciences | 555518 | 1:100 |
| anti-human CD4-PE-Cy7 | BD Biosciences | 560909 | 1:100 |
| anti-human CD8-APC-Cy7. | Biolegend | 300926 | 1:100 |
| Anti-human CD5-FITC | BD Biosciences | 555352 | 1:100 |
| anti-human CD7-APC | Biolegend | 343108 | 1:100 |
| HRP-Conjugated GAPDH Monoclonal Antibody | Proteintech | HRP-60004 | 1:1000 |
| mouse Monoclonal ANTI-FLAG | Sigma | F1804 | 1:1000 |
