# Supplementary material for: Characterization of gene regulatory networks underlying key properties in human hematopoietic stem cell ontogeny
Source: Cell Regen. 2024 Apr 17;13:9. doi: 10.1186/s13619-024-00192-z (PMC11024070; doi:10.1186/s13619-024-00192-z)
Supplement: Supplementary file 3 — Additional file 3: Supplementary Table 2. qRT-PCR primers. [file 13619_2024_192_MOESM3_ESM.pdf]

Supplemental Table 2 qRT-PCR primers

**Primers**	**Sequence (5' – 3')**
GAPDH-PF	GGAGCGAGATCCCTCCAAAAT
GAPDH-PR	GGCTGTTGTCATACTTCTCATGG
HOXA9-PF	TACGTGGACTCGTTCCTGCT
HOXA9-PR	CGTCGCCTTGGACTGGAAG
KLF2-PF	TTCGGTCTCTTCGACGACG
KLF2-PR	TGCGAACTCTTGGTGTAGGTC
KLF9-PF	GCCGCCTACATGGACTTCG
KLF9-PR	GGATGGGTCGGTACTTGTTCA
FOS-PF	CCGGGGATAGCCTCTCTTACT
FOS-PR	CCAGGTCCGTGCAGAAGTC
IKZF1-PF	CATCAGCCCGATGTACCAGC
IKZF1-PR	CCTCGTTGTTGCTCTCGGT
REL-PF	ACATGGTAATTTGACGACTGCT
REL-PR	GCTTCCCAATCGTTCAACACA
